# Transcriptome-based selection and validation of optimal reference genes in perirenal adipose developing of goat (*Capra hircus*)

**DOI:** 10.3389/fvets.2022.1055866

**Published:** 2022-11-17

**Authors:** Le Zhao, Haili Yang, Xingchun Li, Yumei Zhou, Taolu Liu, Yongju Zhao

**Affiliations:** Chongqing Key Laboratory of Herbivore Science, College of Animal Science and Technology, Southwest University, Chongqing, China

**Keywords:** goat, reference genes, brown adipose tissue, whiting, transcriptome

## Abstract

Brown adipose tissue (BAT) is mainly present in young mammals and is important for maintaining body temperature in neonatal mammals because of its ability to produce non-shivering thermogenesis. There is usually a large amount of BAT around the kidneys of newborn kids, but the BAT gradually “whiting” after birth. Screening and validating appropriate reference genes is a prerequisite for further studying the mechanism of goat brown adipose tissue “whiting” during the early stages. In this study, the expression stability of 17 candidate reference genes: 12 *COPS8, SAP18, IGF2R, PARL, SNRNP200, ACTG1, CLTA, GANAB, GABARAP, PCBP2, CTSB*, and *CD151*) selected based on previous transcriptome data as new candidate reference genes, 3 (*PFDN5, CTNNB1*, and *EIF3M*) recommended in previous studies, and 2 traditional reference genes (*ACTB* and *GAPDH*) was evaluated. Real-time quantitative PCR (RT-qPCR) technology was used to detect the expression level of candidate reference genes during goat BAT “whiting”. Four algorithms: Normfinder, geNorm, ΔCt method, and BestKeeper, and two comprehensive algorithms: ComprFinder and RefFinder, were used to analyze the stability of each candidate reference genes. *GABARAP, CLTA, GAPDH*, and *ACTB* were identified as the most stable reference genes, while *CTNNB1, CTSB*, and *EIF3M* were the least stable. Moreover, two randomly selected target genes *IDH2* and *RBP4*, were effectively normalized using the selected most stable reference genes. These findings collectively suggest that *GABARAP, CLTA, GAPDH*, and *ACTB* are relatively stable reference genes that can potentially be used for the development of perirenal fat in goats.

## Introduction

Nowadays, people's over-nutritious diets and sedentary lifestyles are easily causing obesity. Notably, obesity is closely related to hypertension and cardiovascular and metabolic diseases, having long-term health impacts (1). Mammalian adipose tissue can be divided into white adipose tissue (WAT) and brown adipose tissue (BAT) (2, 3). WAT is mainly in the form of triglycerides which store excess energy that is used when needed. BAT can increase the energy consumption of the body, and the adipose tissue has strong plasticity (4). Activating the formation of BAT or converting WAT into BAT could thus be an important strategy for treating obesity in the future (5). BAT also helps protect young animals against cold because it produces non-shivering thermogenesis.

Various techniques and tools, such as whole genome sequencing (WGS), methylated DNA co-immunoprecipitation (MeDIP-Seq), chromatin co-immunoprecipitation (ChIP-seq), and transcriptome sequencing (RNA-seq) have been used to further explore the developmental regulation process of adipose tissue. Quantitative real-time PCR (RT-qPCR) is an important method for analyzing gene expression because of its strong specificity, high sensitivity, and good repeatability. It has thus become a very effective method for detecting gene transcription levels (6–9). However, RT-qPCR results largely depend on the stability of the reference genes (8, 10). The expression of reference genes is not completely universal, and certain differences exist between different tissues, environmental conditions, and species (11–15).

To date, there are only a few systematic studies on goat adipose tissue reference genes despite many scholars having used different algorithms to evaluate some reference genes suitable for human and mouse adipose tissues (12, 16–21). In addition, the internal regulatory mechanism driving the change from BAT to WAT in goat kids remains unclear despite the change process of BAT to WAT occurring in goat and sheep perirenal adipose tissue from birth to adulthood (22, 23). It is particularly important to screen suitable reference genes to further study the internal regulatory mechanism driving this change process.

In this study, we systematically studied the perirenal adipose tissue of Dazu black goats at 0, 7, 14, 21, and 28 d after birth and screened 12 novel candidate reference genes through transcriptome sequencing. The candidate reference genes were compared and ranked using currently available major computational programs geNorm (14), ΔCt (24), Normfinder (25), BestKeeper (26), and RefFinder (27) methods and a comprehensive method ComprFinder (a newly developed method by our team) (10).

## Materials and methods

### Sample collection

Samples were collected at five postnatal stages: 0 days (*n* = 4), 7 days (*n* = 4), 14 days (*n* = 3), 21 days (*n* = 3), and 28 days (*n* = 4), denoted as D0, D7, D14, D21, and D28, respectively. The Dazu black goats were provided by Chongqing Tengda Animal Husbandry Co., Ltd., China.

The perirenal adipose tissue was collected after bloodletting and slaughtering the goats. Part of the perirenal adipose tissues were immediately stored in liquid nitrogen for RNA extraction. The remaining perirenal adipose tissues were washed with sterile saline, preserved in 4% paraformaldehyde, and stored at 4°C for later use in immunohistochemical tests.

### Histological analysis and immunohistochemistry (IHC)

Perirenal adipose tissues were first fixed in 4% neutral buffered formaldehyde (pH 7.4) for over 24 h at room temperature and were then paraffin-embedded and cut into 5 μm sections. The sections were then subjected to hematoxylin-eosin (HE) and IHC staining following standard procedures. The primary antibody (anti-uncoupling protein 1 (UCP1) was purchased from Proteintech Group (Chicago, IL, USA). All images were taken using an Olympus DP73 camera installed on an Olympus IX51 inverted microscope.

### Selection of candidate reference genes

We screened candidate reference genes from the RNA-seq data (Unpublished data) of 18 perirenal adipose tissues in the five stages. The screening of reference genes was based on the coefficient of variation (CV, %) and the fragments per kb per million reads (FPKM) value. The screening criteria were FPKM>50 and CV < 15%.

### RNA extraction and cDNA synthesis

Total RNA from perirenal adipose tissue collected at different stages was extracted using the Trizol reagent (Invitrogen, USA) following the manufacturer's instructions. In brief, the adipose tissues were placed into a centrifuge tube containing 1 mL TRIzol reagent and incubated for 15 min, followed by the addition of 200 mL chloroform (cdkelong, Chengdu, China). The mixture was then centrifuged at 12,000 rpm for 20 min at 4°C to collect the supernatant to which 500 mL isopropanol (cdkelong, Chengdu, China) was added and the mixture further centrifuged at 12,000 rpm for 10 min at 4°C to pellet the RNA. The supernatant was drained off, and the pellet was washed several times with 1 mL of cold 75% ethanol by centrifuging at 12,000 rpm for 5 min at 4°C. The pellets were then air-dried and resuspended in 20 μL of DEPC-treated water. A Nanodrop2000 (ThermoFisher, Meridian, USA) was then used to measure the concentration and optical density (OD) ratio of OD260/OD280 of the RNA. RNA integrity was checked using agarose gel electrophoresis (Bio-Rad, Richmond, USA). First-strand cDNA was synthesized using Prime Script TM RT reagent Kit with gDNA Eraser (Tiangen, China).

### RT-qPCR analysis

The primer pairs of COP9 signalosome subunit 8 (*COPS8*), Sin3A associated protein 18 (*SAP18*), Insulin-like growth factor 2 receptor (*IGF2R*), Small nuclear ribonucleoprotein U5 subunit 200 (*SNRNP200*), Presenilin associated rhomboid like (*PARL*), glucosidase II alpha subunit (*GANAB*), Actin gamma 1 (*ACTG1*), Poly(rC) binding protein 2 (*PCBP*2), Clathrin light chain A (*CLTA*), GABA type A receptor-associated protein (*GABARAP*), Cathepsin B (*CTSB*), CD151 molecule (Raph blood group) (*CD151)*, Prefoldin subunit 5 (*PFDN5*), Catenin beta 1 (*CTNNB1*), Eukaryotic translation initiation factor 3 subunit M (*EIF3M*), Actin beta (*ACTB*) and, Glyceraldehyde-3-phosphate dehydrogenase (*GAPDH*) were designed using the Primer Premier 5.0 software. The sequences of the primer pairs are outlined in Supplementary Table 1. The RT-qPCR reactions were performed on a CFX96 Real-Time System (BIO-RAD) using TB Green ^®^ Premix Ex Taq™ III. The RT-qPCR reaction conditions were: initial denaturation at 95°C for 30s, followed by 40 cycles of denaturation and annealing at 95°C for 5s and 60°C for 30s, respectively. The Ct values were automatically generated using the default settings of the Real-Time System.

### Determination of the expression stability of the candidate reference genes

The evaluation of reference gene expression stability was based of the Ct data from all candidate reference genes obtained from RT-qPCR experiments. It was done using 5 widely used algorithms: geNorm (14), ΔCt (24), Normfinder (25), BestKeeper (26), and RefFinder (27), and the newly developed algorithm ComprFinder (10).

### Validation of selected reference genes

Two genes, *IDH2* which is highly expressed in brown adipose tissue, and *RBP4* which are highly expressed in white adipose tissue, were selected to further verify the effect of the screened reference genes on the normalized target genes. The expression of the target genes was analyzed using traditional, the most stable, and the most unstable reference genes. The relative differences in gene expression were calculated using the 2^−ΔΔCT^ method.

## Results

### RNA-seq-based selection of novel candidate reference genes during perirenal fat development in goats

**Figure 1:**
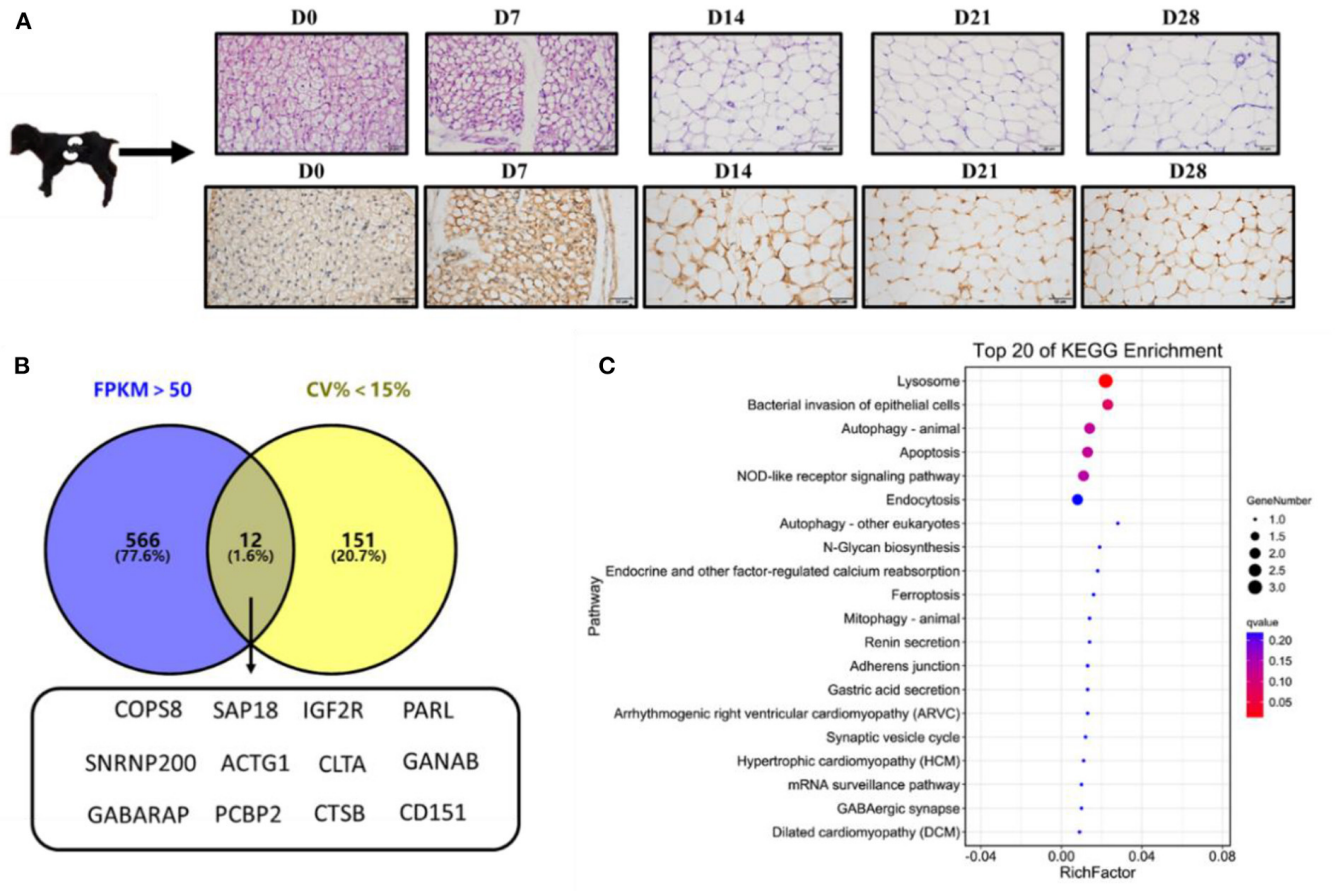
Selection of novel candidate reference genes. **(A)** Hematoxylin-eosin (HE) and UCP1immunohistochemical staining of perirenal adipose tissue; **(B)** Venn diagram of overlapping genes with FPKM >50 and CV < 15%; **(C)** The top 20 enriched signaling pathways of the 12 candidate reference genes based on KEGG analysis.

The histological study of goat perirenal fat showed that the perirenal fat gradually changed from brown adipose tissue at D0 to white adipose tissue at D28 (Figure 1A). The immunohistochemical results of UCP1 also showed that the content of UCP1 was highest at D0, and then gradually decreased (Figure 1A).

Analysis of the transcriptome sequencing data, based on FPKM>50 and CV < 15%, revealed 12 candidate reference genes: *COPS8, SAP18, IGF2R, PARL, SNRNP200, ACTG1, CLTA, GANAB, GABARAP, PCBP2, CTSB*, and *CD151* (Figure 1B). KEGG enrichment analysis revealed that the genes were mainly enriched in lysosome pathways (Figure 1C). Reference genes reported in previous studies, including *PFDN5, CTNNB1*, and *EIF3M* (28), and 2 (*ACTB* and *GAPDH*) traditional reference genes used to study the expression of target genes (10, 29–31) were also included to study the expression levels of target genes in goat perirenal fat. The 17 genes were ranked according to their CV values, with the lower CV values get a higher-ranking order (Supplementary Table 2).

### RNA purity and primer verification of the candidate reference genes

**Figure 2:**
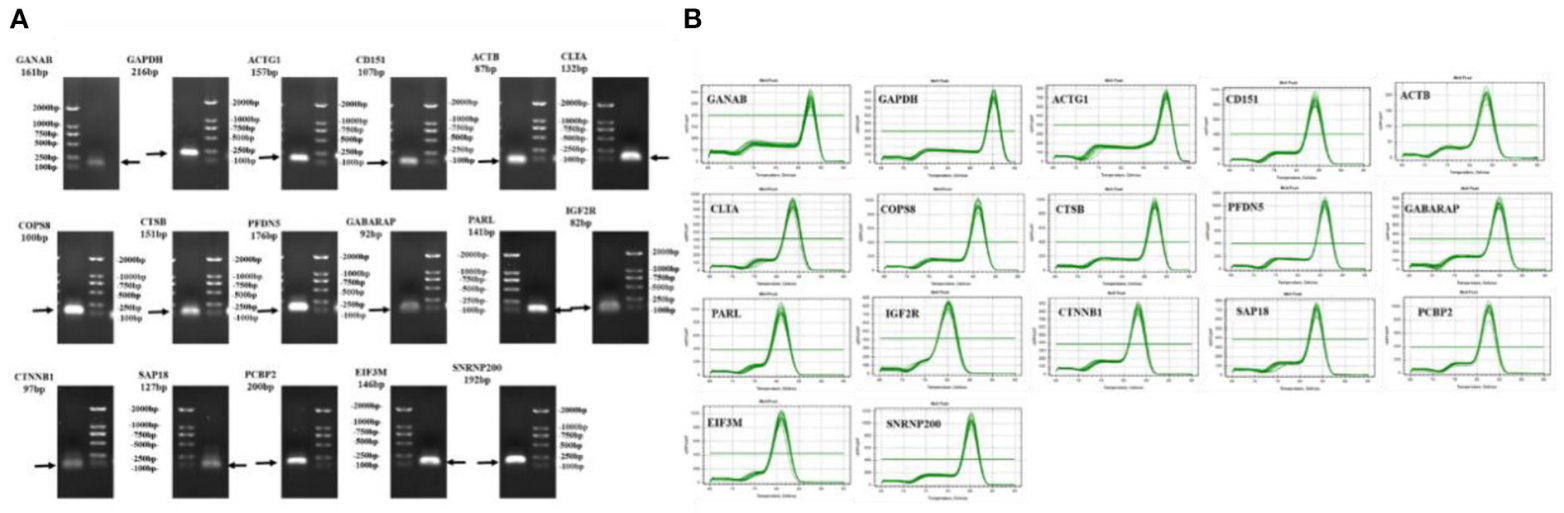
Detection the primer-specific detection of candidate reference genes. **(A)** Agarose gel electrophoresis detection of primers; **(B)** Melting curves of primers.

The RIN values of the 18 RNA samples extracted herein were between 7.6 and 9.7, and their concentrations were also high (Supplementary Table 3), indicating that the RNA quality of the samples was good and could be used for the next experiment. Primer specificity detection results showed that the 17 candidate reference genes had a single melting curve, with no non-specific amplification (Figure 2A). Agarose gel electrophoresis also revealed a single band of the amplified product, suggesting good primer specificity (Figure 2B).

### Analysis of the expression levels of the candidate reference gene

**Figure 3:**
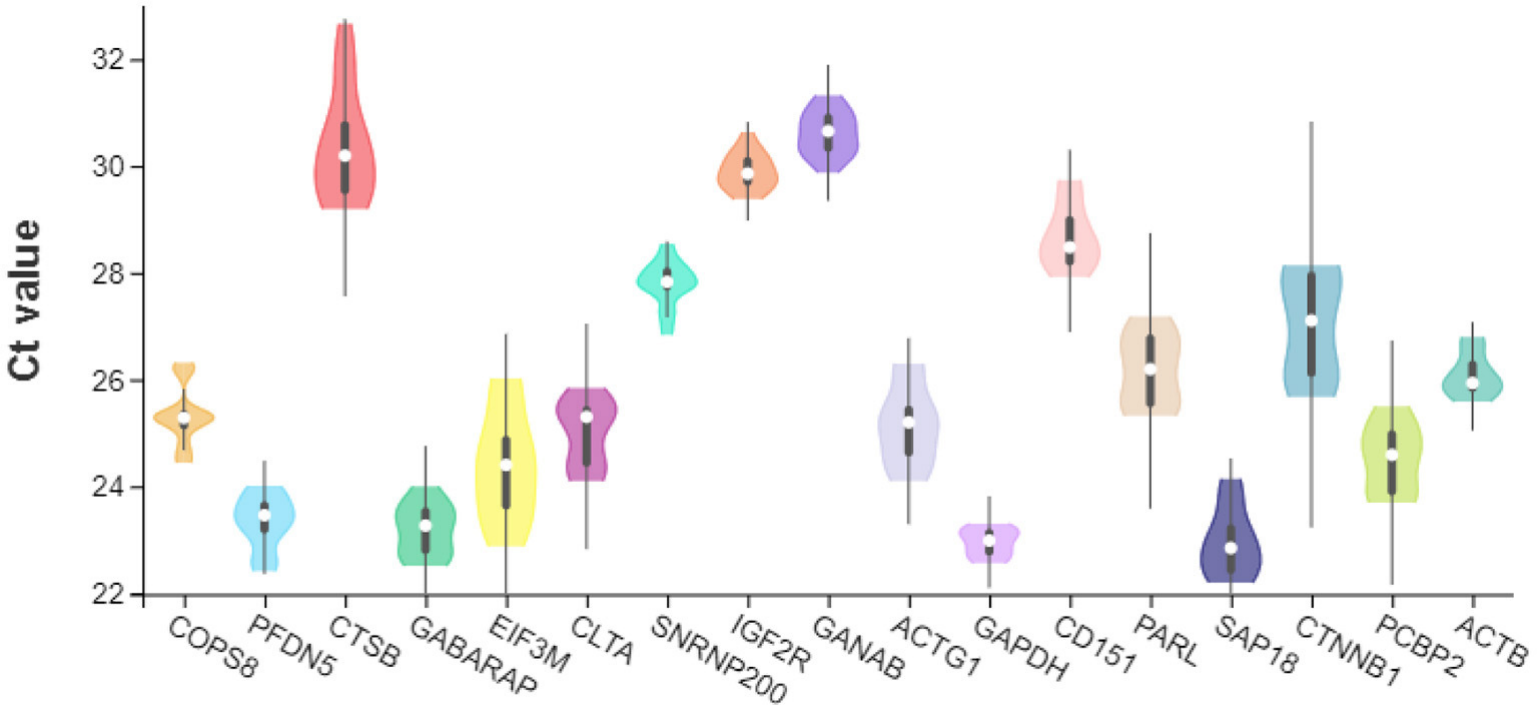
Violin plot of Ct values of 17 candidate reference genes.

The expression levels of the 17 candidate reference gene were tested by qPCR. Notably, *SAP18* (mean Ct value: 22.938) had the lowest cycle threshold (Ct) value, while *GANAB* (mean Ct value: 30.617) had the highest Ct (Figure 3). The Ct values of the other genes lay within certain ranges: *GAPDH* 22.56 and 23.30 (mean Ct value: 22.9535), *GABARAP* 22.51 and 24.00 (mean Ct value: 23.187), *PFDN5* 22.41 and 24.01 (mean Ct value: 23.3385), *EIF3M* 22.87 and 26.02 (mean Ct value: 24.3755), *PCBP2* 23.69 and 25.51 (mean Ct value: 24.5305), *CLTA* 24.09 and 25.85 (mean Ct value: 25.0085), *ACTG1* 24.09 and 26.31 (mean Ct value: 25.147), *COPS8* 24.44 and 26.32 (mean Ct value: 25.3245), *ACTB* 25.59 and 26.80 (mean Ct value: 26.089), PARL 25.31 and 27.19 (mean Ct value: 26.186), *CTNNB1* 25.68 and 28.15 (mean Ct value: 26.186) 27.0155, *SNRNP200* 26.83 and 28.55 (mean Ct value: 27.797), *CD151* 27.92 and 29.75 (mean Ct value: 28.687), *IGF2R* 29.38 and 30.63 (mean Ct value: 29.8755), *CTSB* 29.19 and 32.67 (mean Ct value: 30.363), and *GANAB* 29.88 and 31.32 (mean Ct value: 30.617). *GABARAP, GANAB, IGF2R, ACTB*, and *GAPDH* had the most stable Ct values, while the *CTSB, EIF3M*, and *CTNNB1* had the most unstable Ct values.

### GeNorm analysis

**Figure 4:**
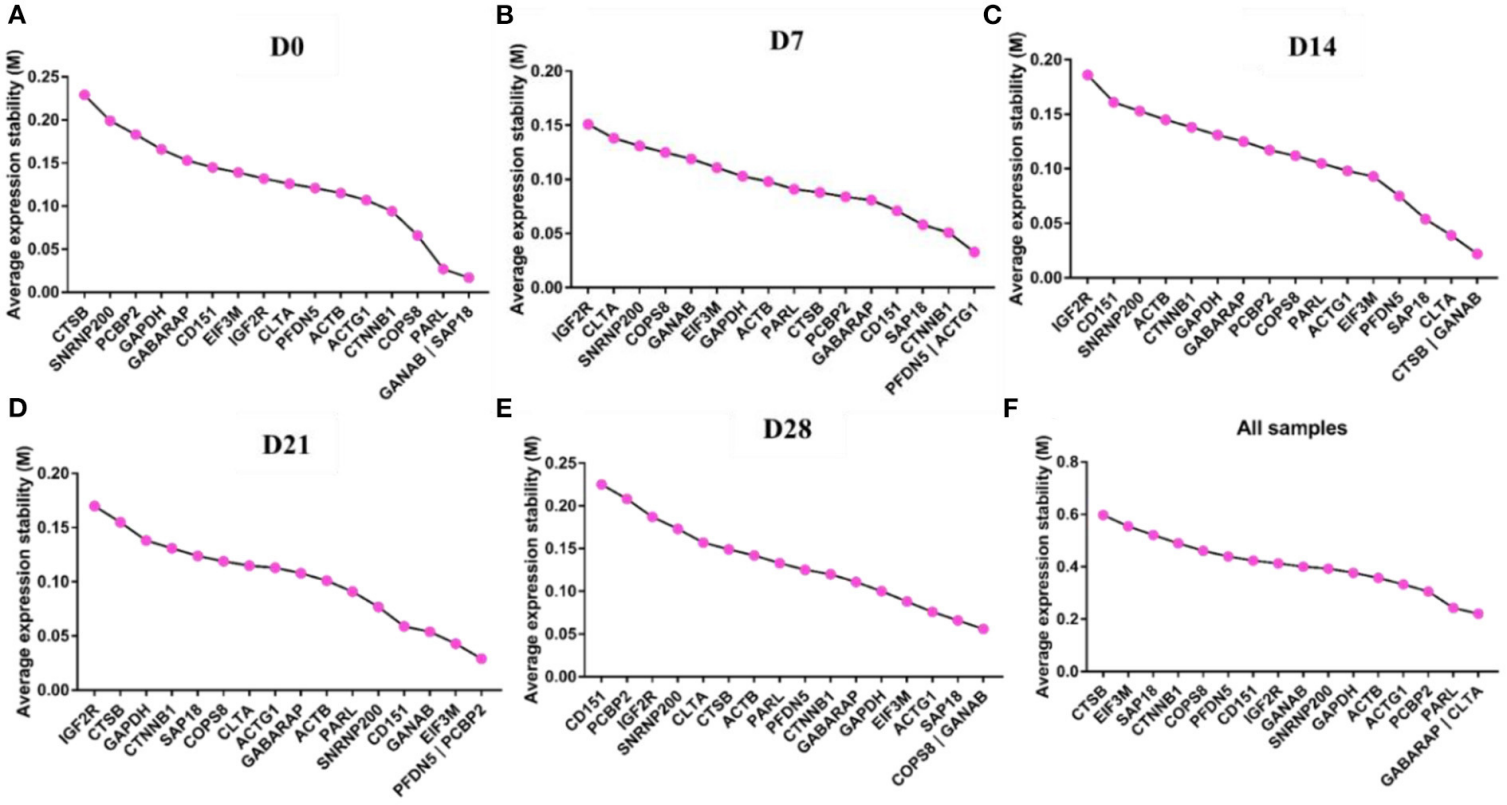
GeNorm analysis the average expression stability of candidate reference genes. **(A)** D0, **(B)** D7, **(C)** D14, **(D)** D21, **(E)** D28, and **(F)** All samples including D0–D28.

GeNorm was used to analyze the expression stability of the 17 candidate reference genes in goat perirenal fat at different periods after birth. The M value reflected the gene expression stability; the lower the M value, the more stable the gene expression and vice versa. *GANAB* and *SAP18* had the lowest M values, while *CTSB* had the highest M values at D0 (Figure 4A). *PFDN5* and *ACTG1* were the most stable genes, while *IGF2R* was the least stable at D7 (Figure 4B). *CTSB* and *GANAB* were the most stable genes, while IGF2R was the least stable gene at D14 (Figure 4C). *PFDN5* and *PCBP2* were the most stable genes, while *IGF2R* was the least stable gene at D21 (Figure 4D). *COPS8* and *GANAB* were the most stable genes, while *CD151* was the least stable gene at D28 (Figure 4E). The rank order of all samples based on the M value was: CTSB>*EIF3M*>*SAP18*>*CTNNB1*>*COPS8*>*PFDN5*>*CD151*>*IGF2R*>*GANAB*>*SNRNP200*>*GAPDH*>*ACTB*>*ACTG1*>*PCBP2*>*PARL*> *GABARAP* and *CLTA* (Figure 4F). In the pairwise variation analysis, we found all the experimental variables were below the cut-off value of 0.15 (Supplementary Figure 1).

### Normfinder analysis

**Figure 5:**
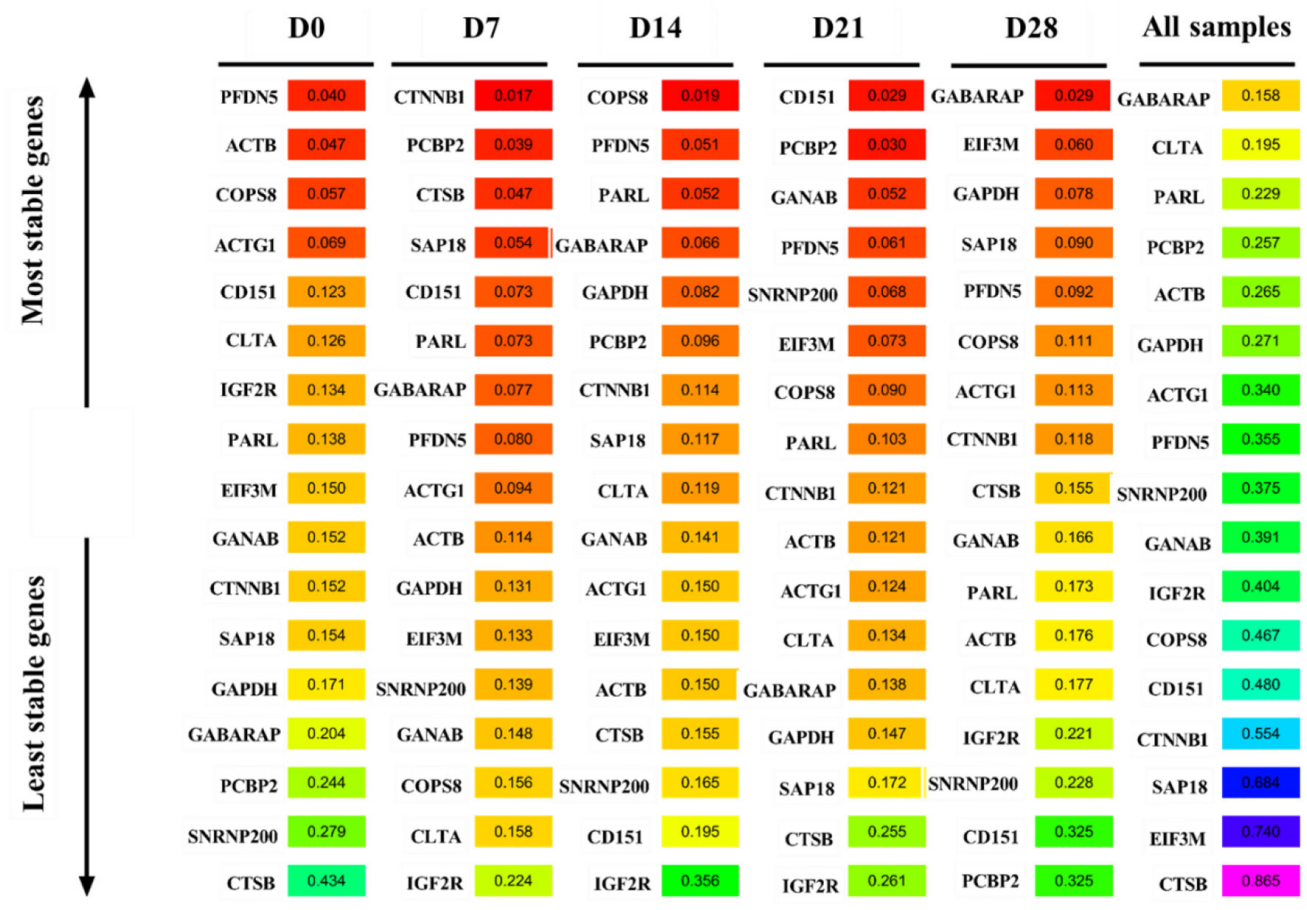
Normfinder analysis the average expression stability of candidate reference genes.

Figure 5 shows the Normfinder-based analysis results of the expression stability of the 17 candidate reference genes. *PFDN5* was the most stable gene, while *CTSB* was the least stable gene at D0. *CTNNB1, COPS8*, and *CD151* were the most stable genes at D7, D14, and D21, respectively, while *IGF2R* was the most unstable gene. *GABARAP* was the most stable gene, while *PCBP2* was the most unstable gene at D28. Notably, *GABARAP* was the most stable gene, while *CTSB* was the most unstable gene in all samples.

### BestKeeper analysis

**Figure 6:**
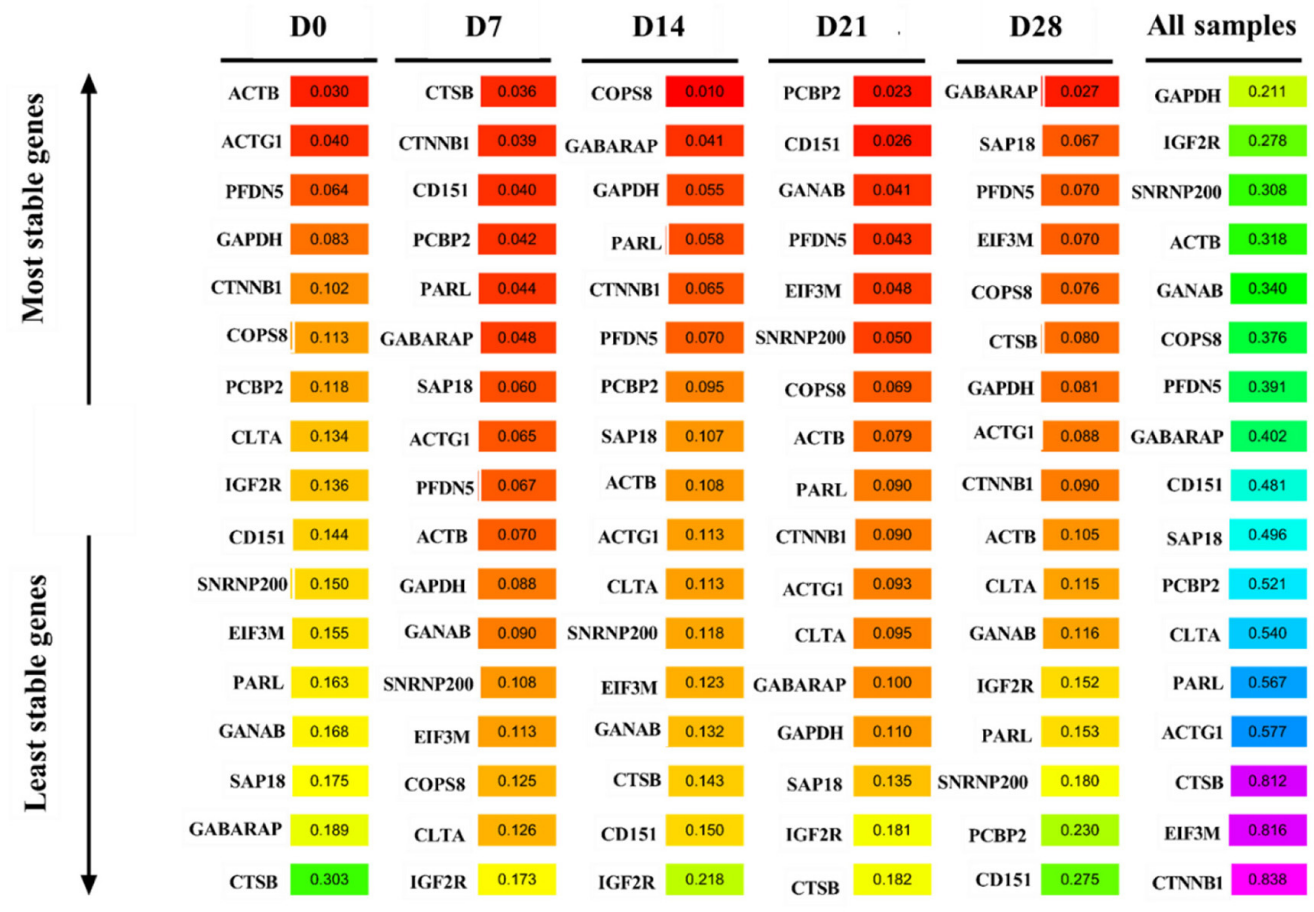
BestKeeper analysis the expression stability of candidate reference genes.

The stability of the candidate reference genes was also assessed using the BestKeeper; the lower the std-value, the more stable the gene expression, and vice versa. *ACTB* and *PCBP2* were the most stably expressed genes at D0 and D21, respectively (Figure 6). In contrast, *CTSB* was the most unstable gene at D0 and D21but the most stable gene at D7. *IGF2R* was the most unstable gene at D7 and D14. *GABARAP* was the most stable gene, while *CD151* was the most unstable gene at D28. Notably, *GAPDH, IGF2R, SNRNP200, ACTB, GANAB, COPS8, PFDN5*, and *GABARAP* were the most stable genes, while *CTNNB1, EIF3M*, and *CTSB* were the most unstable genes in all samples.

### ΔCt analysis

**Figure 7:**
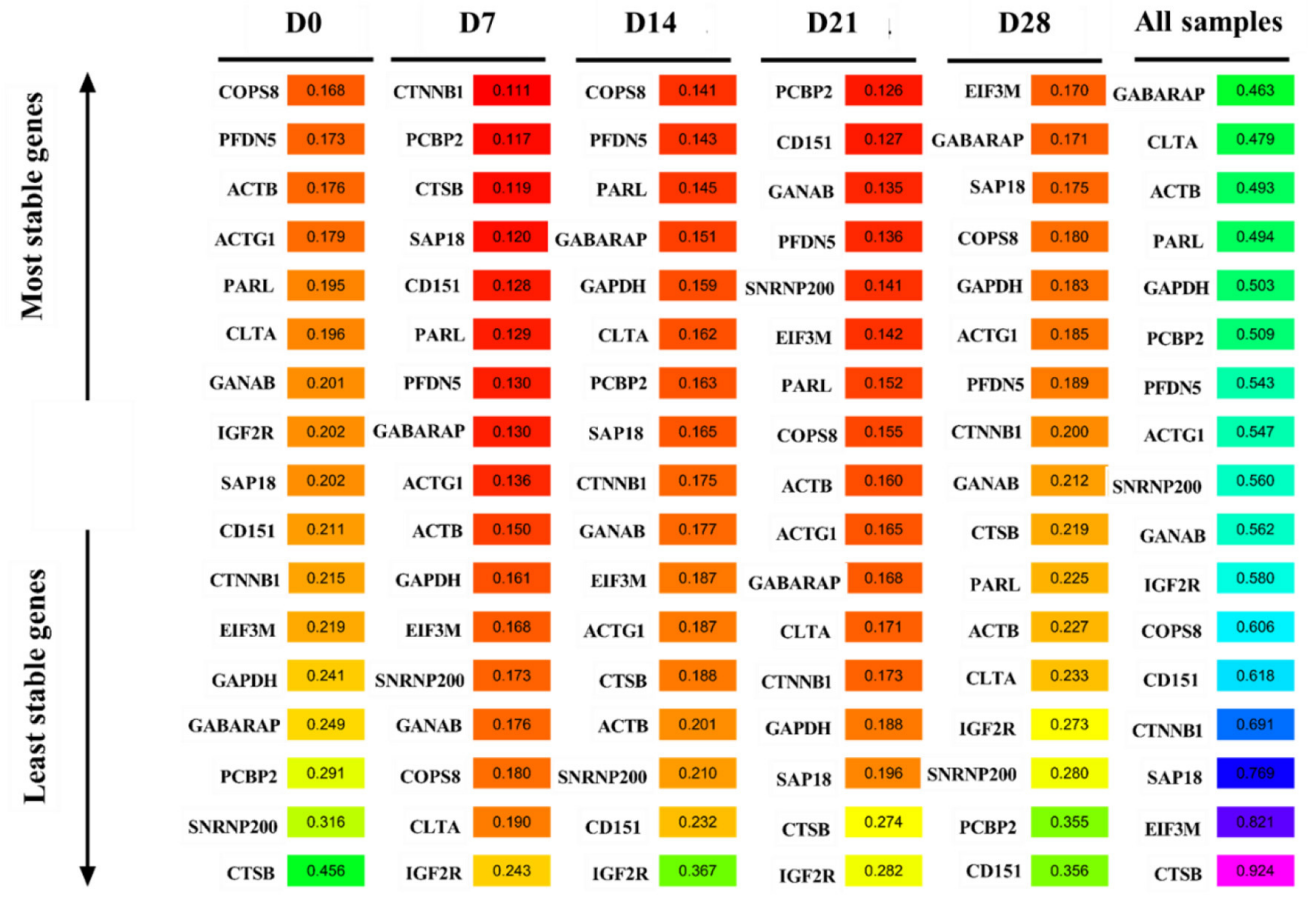
ΔCt method analysis the expression stability of candidate reference genes.

Figure 7 shows the analysis results of the expression stability of the 17 candidate reference genes based on the ΔCt method. *COPS8* was the most stable gene at D0 and D14, while *CTSB* was the least stable gene at D0. *CTTNB1* and *PCBP2* were the most stably expressed genes at D7 and D21, respectively. In contrast, *IGF2R* was the most unstable gene at D7, D14, and D21. *EIF3M* was the most stable gene, while *CD151* was the most unstable gene at D28. *GABARAP* was the most stable gene, while *CTSB* was the most unstable gene in all samples.

### RefFinder analysis

**Figure 8:**
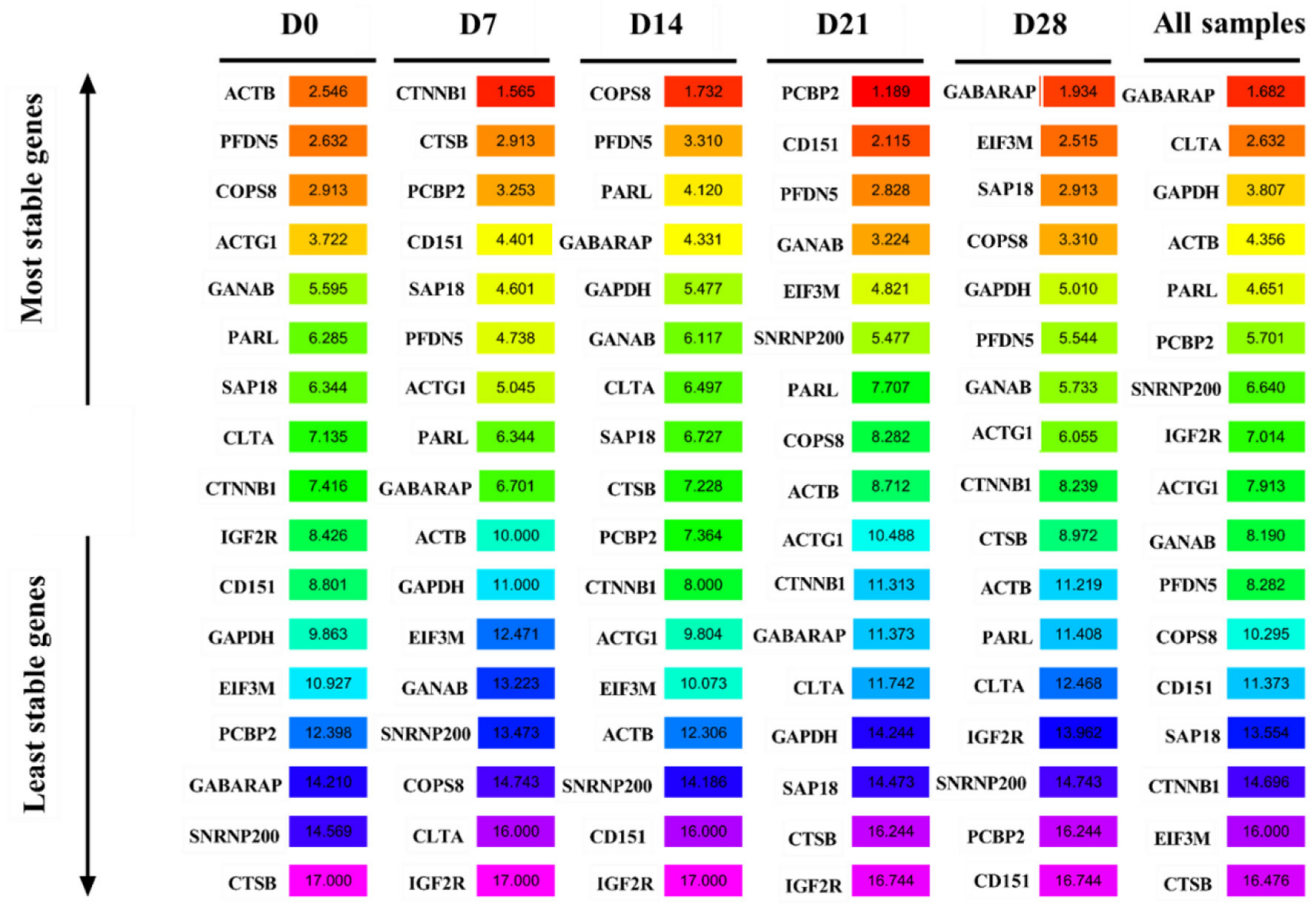
RefFinder analysis the expression stability of candidate reference genes.

The RefFinder algorithm was used to comprehensively rank the candidate reference genes based on geNorm, Normfinder, BestKeeper, and ΔCt methods. *ACTB* was the most stable gene, while *CTSB* was the most unstable gene at D0. *CTTNB1, COPS8*, and *PCBP2*were the most stably expressed genes at D7, D14, and D21, respectively. In contrast, *IGF2R* was the most unstable gene at D7, D14, and D21. *GABARAP* was the most stable gene, while *CD151* was the most unstable gene at D28. *GABARAP* was the most stable gene, while *CTSB* was the most unstable gene in all samples (Figure 8).

### ComprFinder analysis

**Figure 9:**
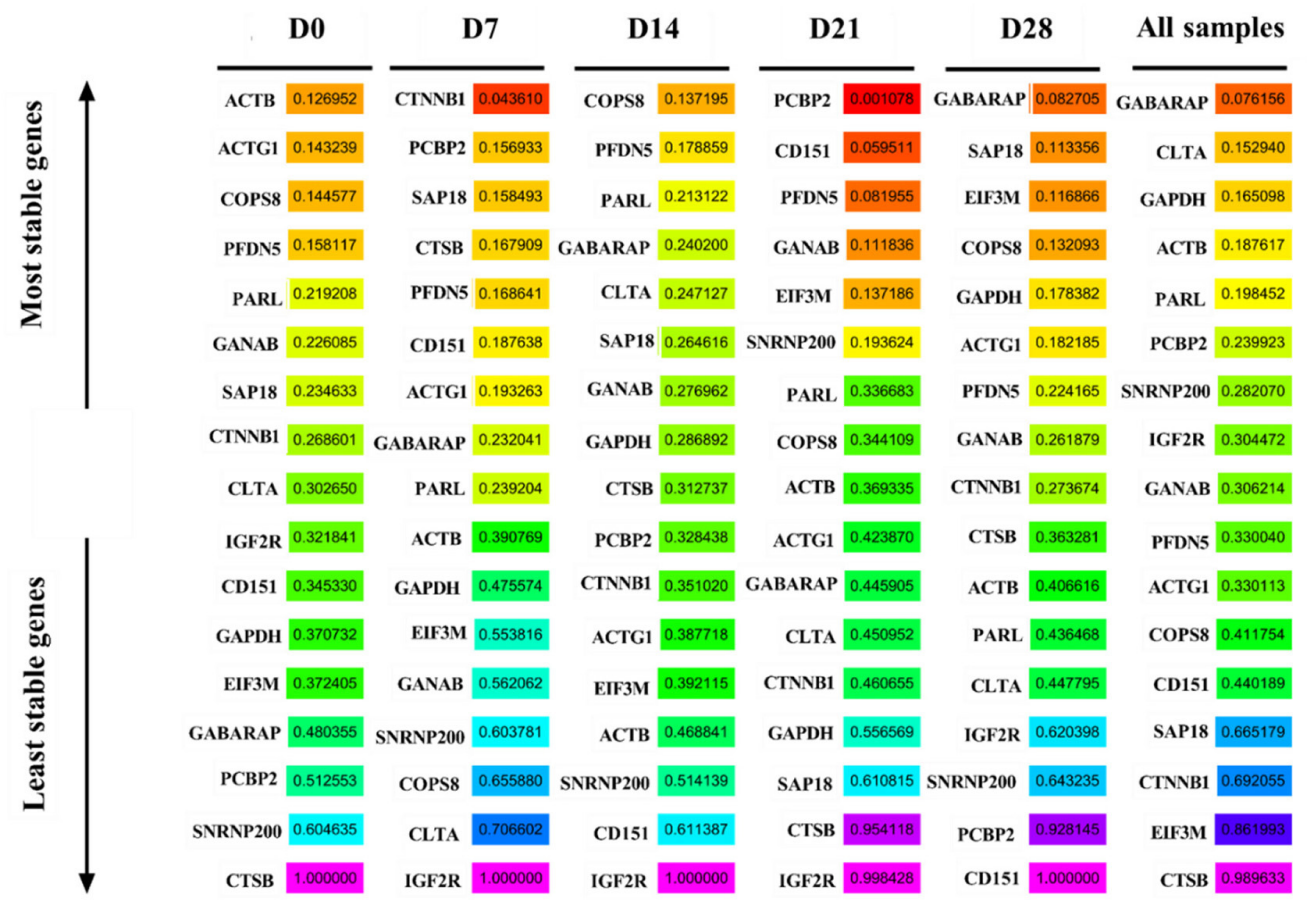
ComFinder analysis the expression stability of candidate reference genes.

ComprFinder is a new comprehensive analysis algorithm developed by our team during the early stage. Figure 9 shows the ComprFinder-based analysis results of the expression stability of the 17 candidate reference genes. *ACTB* was the most stable gene, while *CTSB* was the most unstable gene at D0. *CTTNB1, COPS8*, and *PCBP2*were the most stably expressed genes at D7, D14, and D21, respectively. However, *IGF2R* was the most unstable gene at D7, D14, and D21. *GABARAP* was the most stable gene, while *CD151* was the most unstable gene at D28. *GABARAP* was the most stable gene, while *CTSB* was the most unstable gene in all samples.

### Validation of the most stable reference genes with target genes

**Figure 10:**
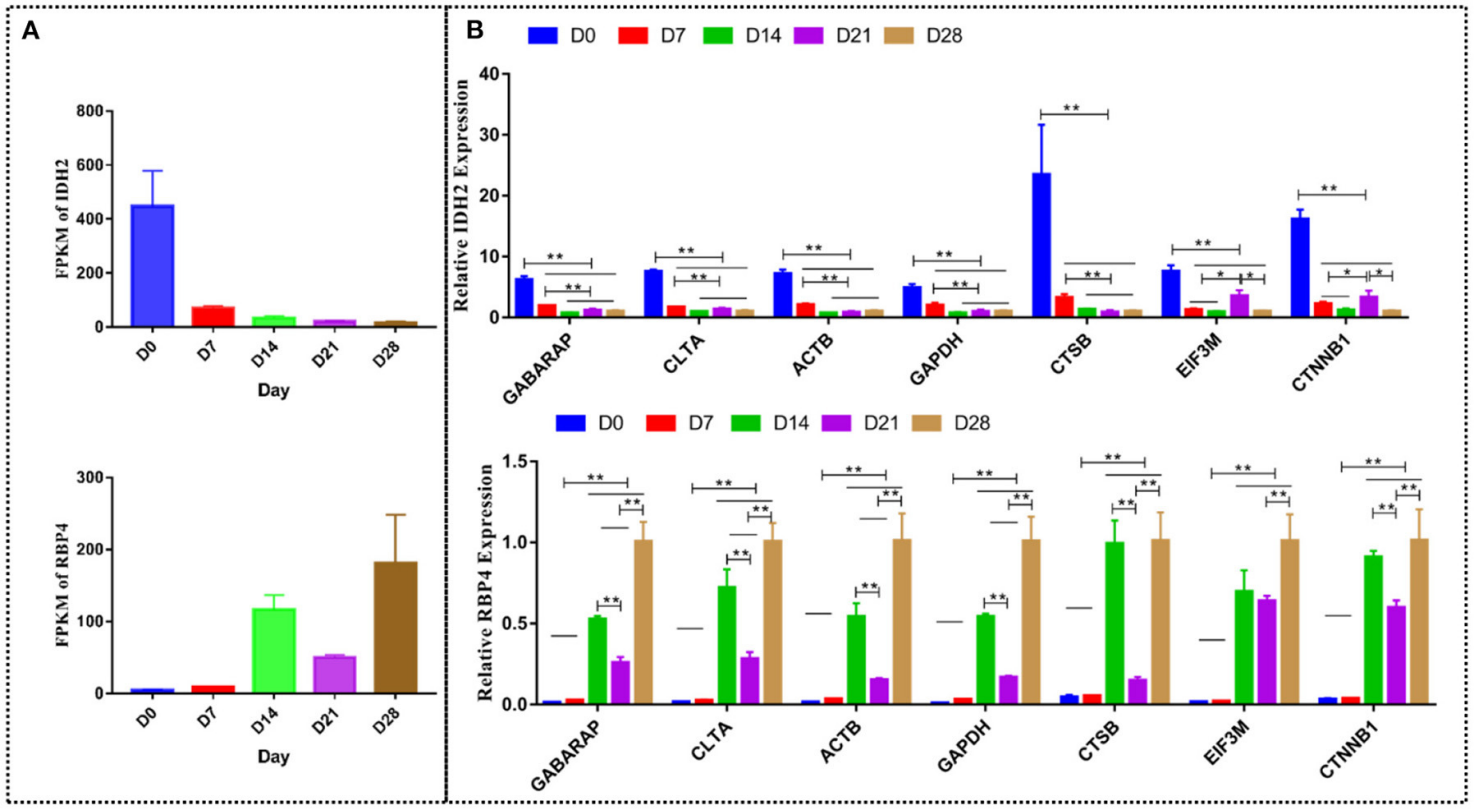
The relative expression of *IDH2* and *RBP4* normalized by different reference genes. **(A)** The mRNA expression level measured by RNA-seq. **(B)** The expression of *IDH2* and *RBP4* was normalized using *GABARAP, CLTA, ACTB, GAPDH, CTSB, EIF3M*, and *CTNNB1* genes. *p < 0.05; **p < 0.01.

*GABARAP* was the most stable gene, while *CTSB* was the most unstable gene amongst the 17 candidate reference genes at different stages of goat development. Similar target genes were normalized using the most stable reference genes *GABARAP* and *CLTA*, the traditional reference genes *ACTB* and *GAPDH*, and the most unstable reference genes *CTSB, EIF3M*, and *CTNNB1* to further validate the candidate reference genes. The gene expression levels of *IDH2* and *RBP4* were consistent with the RNA-seq data. *IDH2* was highly expressed at D0 (brown adipose tissue), while RBP4 was highly expressed at D28 (white adipose tissue). The expression of *IDH2* at D0 was significantly higher than at D7 (*P* < 0.01), D14 (*P* < 0.01), D21 (*P* < 0.01), and D28 (*P* < 0.01), while its expression at D7 was significantly higher than at D14 (*P* < 0.01), D21 (*P* < 0.01), and D28 (*P* < 0.01) when *IDH2* was normalized with *GABARAP, CLTA, ACTB, GAPDH*, and *CTSB*. In the same line, the expression of *IDH2* at D0 was significantly higher than at D7 (*P* < 0.01), D14 (*P* < 0.01), D21 (*P* < 0.01), and D28 (*P* < 0.01), while its expression at D21 was significantly higher than at D7 (*P* < 0.05), D14 (*P* < 0.05) and D28 (*P* < 0.05) when *IDH2* was normalized with *EIF3M* and *CTNNB1*. The expression of *RBP4* at D28, D21, and D14 was significantly higher than at D0 (*P* < 0.01) and D7 (*P* < 0.01), its expression at D28 was significantly higher than at D14 (*P* < 0.01) and D21 (*P* < 0.01), while its expression at D21 was significantly lower than D14 (*P* < 0.01) when *RBP4* was normalized with *GABARAP, CLTA, ACTB, GAPDH, CTSB*, and *CTNNB1*. The expression of *IDH2* at D28, D21, and D14 was significantly higher than at D0 (*P* < 0.01) and D7 (*P* < 0.01), while its expression at D28 was significantly higher than at D21 (*P* < 0.01) when *IDH2* was normalized with *EIF3M* (Figure 10). Of note, the target genes exhibited varying statistical differences when different reference genes were used, highlighting the importance of selecting appropriate reference genes.

## Discussion

Goat BAT is mainly observed around the kidney at birth. In this study, BAT rapidly “whiting” within 2 weeks and turns into WAT at about 4 weeks, which was consistent with the results of previous studies in goats and sheep (22, 23, 28). Transcriptome sequencing is an important research method for gene expression analysis and screening differentially expressed and functional genes. Notably, screening reference genes using transcriptome data is an effective experimental method for screening reference genes in non-model species (32–34). *RPS4X* and *RPS6* are more stable than traditionally used housekeeping genes in the goat rumen (35), while *NCBP3, SDHA*, and *PTPRA* are more stable than traditionally used housekeeping genes in goat skin tissue (10).

Adipose tissue has strong plasticity and is easily affected by environmental temperature, diet, and hormones. Some scholars have studied the stability of reference genes in adipose tissue. For instance, *WDR33* and *HDAC3* are relatively stable reference genes in bovine adipose tissue (13), *TOP2B* and *UXT* in buffalo adipose tissue (36), and *TBP* in mice (16). Herein, *GABARAP* was the most stable reference gene, followed by *CLTA. GABARAP* has also been reported to be a more stable reference gene in ovine pulmonary adenocarcinoma (32). At the same time, *CLTA* is a relatively stable reference gene in melanoma samples and melanoma cell lines (37). *ACTB* and *GAPDH* are traditional reference genes but also showed better expression stability in this study, a finding that was consistent with previous reports. For example, *ACTB* has been postulated to be the most suitable reference gene in the 3T3-L1 adipocyte differentiation model (38). *ACTB* exhibits medium stability as a reference gene in goat perirenal adipose (28). In this study, *ACTG1* exhibited medium stability. However, *ACTG1* presents less stable expression when employed as a reference gene for cerebral cortical astrocytes (39). A previous study postulated that *CTNNB1, PFDN5*, and *EIF3M* are the most stable reference genes for BAT to WAT in goats (28). However, *PFDN5* showed medium stability, while *CTNNB1* and *EIF3M* had poor stability in this study. This variance was attributed to the previous study collecting tissues at three postnatal periods (1 day, 30 days, and 1 year after birth), covering the entire growth cycle but with a larger period. In contrast, this study concentrated on the early growth stage, picking samples at five postnatal stages (0, 7, 14, 21, and 28 days). The differences in the results further indicate that the same reference gene may have large transcriptional differences in samples under different conditions. It also emphasizes the proper selection of appropriate reference genes because it directly impacts the research results.

*RBP4* is a useful biomarker for diagnosing obesity and the prognosis of related diseases (40), while *IDH2* affects brown adipose tissue thermogenesis (41). In this study, *RBP4* and *IDH2* were differentially expressed based on the RNA-seq data (data not shown). However, the results differed when *RBP4* and *IDH2* were normalized using different reference genes. Therefore, selecting reference genes with relatively stable expressions under different conditions can more accurately quantify the expression of target genes in different samples.

## Conclusion

*GABARAP, CLTA, GAPDH*, and *ACTB* genes are relatively stable reference genes that can potentially be used to develop perirenal fat in goats.

## Data availability statement

The data presented in the study are deposited in the Genome Sequence Archive (Genomics, Proteomics and Bioinformatics 2021) in National Genomics Data Center (Nucleic Acids Res 2022), accession number CRA008594.

## Ethics statement

All animal experiments followed the Southwest University Institutional Animal Care and Use Committee (22-9-2019, No. GB14925-2010) regulations. Written informed consent was obtained from the owners for the participation of their animals in this study.

## Author contributions

LZ drafted the manuscript. LZ, XL, YZho, and TL collected experimental tissues. LZ, HY, and XL collected the data and organized the references. YZha was involved in this study design and wrote the manuscript. All authors contributed to the article and approved the submitted version.

## Funding

This work was financially supported by the Chongqing's Modern Agricultural Industry Technology System Program for Herbivore [2022(12)], the Collection, Utilization and Innovation of Germplasm Resources by Research Institutes and Enterprises of Chongqing, China (cqnyncw-kqlhtxm), the National Natural Science Foundation of China (No. 31772564), the Chongqing Postgraduate Research Innovation Project (CYB22141).

## Conflict of interest

The authors declare that the research was conducted in the absence of any commercial or financial relationships that could be construed as a potential conflict of interest.

## Publisher's note

All claims expressed in this article are solely those of the authors and do not necessarily represent those of their affiliated organizations, or those of the publisher, the editors and the reviewers. Any product that may be evaluated in this article, or claim that may be made by its manufacturer, is not guaranteed or endorsed by the publisher.
