# Supplementary material for: Transcriptome-based selection and validation of optimal reference genes in perirenal adipose developing of goat (Capra hircus)
Source: Front Vet Sci. 2022 Nov 17;9:1055866. doi: 10.3389/fvets.2022.1055866 (PMC9712442; doi:10.3389/fvets.2022.1055866)
Supplement: Supplementary file 1 [file Data_Sheet_1.zip › SupMaterial/Revised supplementary table.docx]

SUPPLEMENTARY TABLES

TABLE. S1 Primers used for RT-qPCR

| Gene_name | Gene ID | Primer sequence（5'→3'） | Product size /bp | Primer efficiency (%) |
| --- | --- | --- | --- | --- |
| *COPS8* | ENSCHIG00000026994 | F: GGTGATGGCGGAAAGTTCT | 100 | 108.50% |
|  |  | R: ATACACTGCGGGTGTTGCA |  |  |
| *SAP18* | ENSCHIG00000016828 | F: GGCCTGGCTATCGAGTAAAG | 127 | 95.90% |
|  |  | R: GGAGGGGTAATCGCTATGTC |  |  |
| *IGF2R* | ENSCHIG00000010830 | F: CTTGTTCTACACTTCGGAGGC | 82 | 106.80% |
|  |  | R: CTTATTACAGATGGGCACTTGG |  |  |
| *SNRNP200* | ENSCHIG00000020611 | F: GATAAGGCTCAACGAACCAAAC | 192 | 91.40% |
|  |  | R: CAGCAGCACTTCATAGGTCTCC |  |  |
| *PARL* | ENSCHIG00000026220 | F: AGAAGAAGCAGCCTTTTACCC | 141 | 102.9% |
|  |  | R: CTTTGGACCTTGGATTTCAGTG |  |  |
| *GANAB* | ENSCHIG00000022447 | F: TTGAGCGGGTAGTGATAATAGG | 161 | 90.80% |
|  |  | R: GTGAATAGTCCAGTCAGATGCC |  |  |
| *ACTG1* | ENSCHIG00000026676 | F: CCACTGGCATTGTCATGGAC | 157 | 95.60% |
|  |  | R: AAGCTGTAACCACGTTCCGT |  |  |
| *PCBP2* | ENSCHIG00000019895 | F: CGGAGTGATTGAAGGTGGAT | 200 | 93.40% |
|  |  | R: TGAAGATGGCATTAGTGGGT |  |  |
| *CLTA* | ENSCHIG00000016030 | F: GAGTAATGGGCCGACAGACA | 132 | 104.50% |
|  |  | R: TTCCGAGAATTGGCATCAAG |  |  |
| *GABARAP* | ENSCHIG00000021470 | F: TAAAGAGGAGCATCCGTTCG | 92 | 96.70% |
|  |  | R: CCTTTTCTACTATCACCGGGAC |  |  |
| *CTSB* | ENSCHIG00000025426 | F: CCTCTATGACTCGCATGTAGGTT | 151 | 91.6% |
|  |  | R: GTCTTTGTAGGACGGGCTGTA |  |  |
| *CD151* | ENSCHIG00000008816 | F: GCCTGCTCCTTATCATCTTTC | 107 | 97.90% |
|  |  | R: CATGGTGTCCTTGAGGTTCTC |  |  |
| *PFDN5* | ENSCHIG00000020918 | F: GAATCTGCCGCAGCTAGAAA | 176 | 96.30% |
|  |  | R: AACTCGTCAGTGGGACAAGTAA |  |  |
| *CTNNB1* | ENSCHIG00000025557 | F: CAGCGTCGTACATCTATGGG | 97 | 97.90% |
|  |  | R: GGATGTGAAGGGCTCCAGTA |  |  |
| *EIF3M* | ENSCHIG00000012027 | F: ATGGATTTCTGACTGGAACCTC | 146 | 96.30% |
|  |  | R: AAGCATTGTCCTCCGTGTAA |  |  |
| *ACTB* | ENSCHIG00000013316 | F: CCTGCGGCATTCACGAAACTAC | 87 | 96.20% |
|  |  | R: ACAGCACCGTGTTGGCGTAGAG |  |  |
| *GAPDH* | ENSCHIG00000022516 | F: TTATGACCACTGTCCACGCC | 216 | 90.00% |
|  |  | R: TCAGATCCACAACGGACACG |  |  |
| *IDH2* | ENSCHIG00000003808 | F: GAGAAGCACTACAAGACCGAGTT | 184 | 99.4% |
|  |  | R: ACGTCATCAGACCAAGGGAG |  |  |
| *RBP4* | ENSCHIG00000023599 | F: GAAAGGAAACGATGACCACTG | 122 | 109.3% |
|  |  | R: GGGCGAACACGAAAGAGTAG |  |  |

**TABLE. S2** The summarised information of 17 potential reference genes using RNA-Seq Data

| Type | Ensembl_ID | Gene_name | Mean_FPKM | SD | CV | CV% |
| --- | --- | --- | --- | --- | --- | --- |
| New predicted candidate HKG | ENSCHIG00000026994 | *COPS8* | 51.861 | 4.816 | 0.093 | 9.286 |
|  | ENSCHIG00000016828 | *SAP18* | 79.015 | 7.862 | 0.100 | 9.950 |
|  | ENSCHIG00000010830 | *IGF2R* | 53.719 | 5.593 | 0.104 | 10.412 |
|  | ENSCHIG00000020611 | *SNRNP200* | 61.914 | 6.709 | 0.108 | 10.836 |
|  | ENSCHIG00000026220 | *PARL* | 58.402 | 7.160 | 0.123 | 12.260 |
|  | ENSCHIG00000022447 | *GANAB* | 55.885 | 6.904 | 0.124 | 12.355 |
|  | ENSCHIG00000026676 | *ACTG1* | 946.395 | 120.409 | 0.127 | 12.723 |
|  | ENSCHIG00000019895 | *PCBP2* | 97.527 | 12.720 | 0.130 | 13.043 |
|  | ENSCHIG00000016030 | *CLTA* | 57.215 | 7.569 | 0.132 | 13.229 |
|  | ENSCHIG00000021470 | *GABARAP* | 105.389 | 14.481 | 0.137 | 13.740 |
|  | ENSCHIG00000025426 | *CTSB* | 94.906 | 13.873 | 0.146 | 14.618 |
|  | ENSCHIG00000008816 | *CD151* | 85.937 | 12.834 | 0.149 | 14.934 |
| Suggested by previous study | ENSCHIG00000020918 | *PFDN5* | 45.071 | 8.495 | 0.188 | 18.848 |
|  | ENSCHIG00000025557 | *CTNNB1* | 55.202 | 15.730 | 0.285 | 28.496 |
|  | ENSCHIG00000012027 | *EIF3M* | 54.257 | 21.010 | 0.387 | 38.722 |
| Conventional HKGs | ENSCHIG00000013316 | *ACTB* | 1.597 | 0.783 | 0.490 | 49.027 |
|  | ENSCHIG00000022516 | GAPDH | 724.079 | 403.683 | 0.558 | 55.751 |

**TABLE. S3** Detection the RNA quality of all samples

| Sample | Concentration (ng/μL) | Volume (μL) | Total (μg) | RIN Value |
| --- | --- | --- | --- | --- |
| D0-1 | 1038 | 35 | 36.33 | 9.4 |
| D0-2 | 1686 | 35 | 59.01 | 9.4 |
| D0-3 | 1026 | 40 | 41.04 | 9.7 |
| D0-4 | 957 | 38 | 36.37 | 9 |
| D7-1 | 1062 | 35 | 37.17 | 8.5 |
| D7-2 | 438 | 40 | 17.52 | 9.1 |
| D7-3 | 801 | 35 | 28.04 | 8.7 |
| D7-4 | 723 | 39 | 28.20 | 9.1 |
| D14-1 | 537 | 34 | 18.26 | 9.4 |
| D14-2 | 430 | 39 | 16.77 | 9.1 |
| D14-3 | 434 | 37 | 16.06 | 8.5 |
| D21-1 | 454 | 36 | 16.34 | 8.6 |
| D21-2 | 311 | 34 | 10.57 | 7.6 |
| D21-3 | 396 | 37 | 14.65 | 9.2 |
| D28-1 | 304 | 35 | 10.64 | 8.3 |
| D28-2 | 216 | 38 | 8.21 | 8.7 |
| D28-3 | 405 | 36 | 14.58 | 8.7 |
| D28-4 | 357 | 36 | 12.85 | 8.7 |
