# Supplementary figures and images for: Transcriptome-based selection and validation of optimal reference genes in perirenal adipose developing of goat (Capra hircus)
Source: Front Vet Sci. 2022 Nov 17;9:1055866. doi: 10.3389/fvets.2022.1055866 (PMC9712442; doi:10.3389/fvets.2022.1055866)

**SUPPLEMENTARY FIGURE**

**Figure. S1** The pairwise variation in 17 reference genes by geNorm.


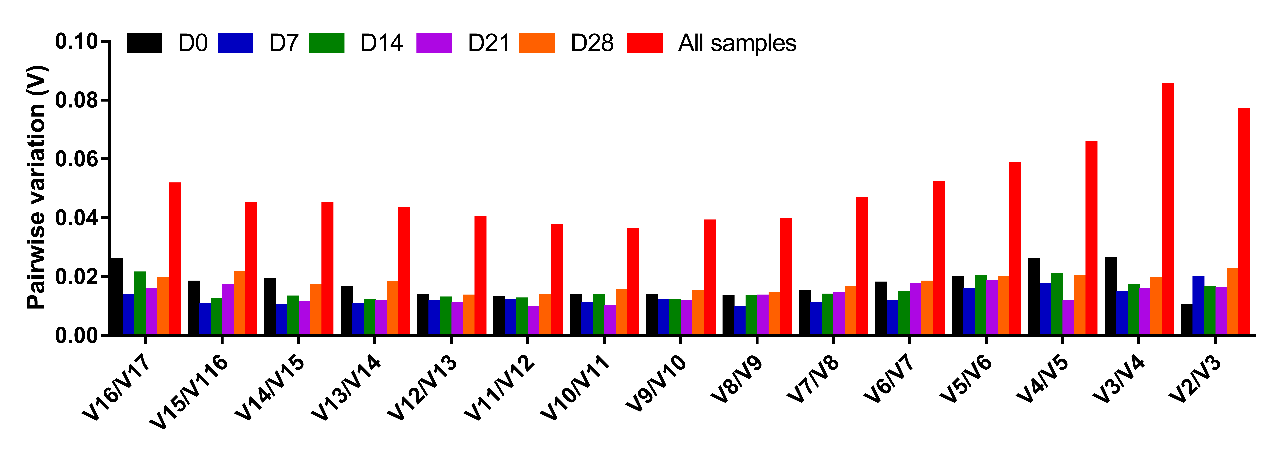

Supplement: Supplementary file 1 [file Data_Sheet_1.zip › SupMaterial/Revised supplementary figure.docx]
